# Synthetic Cannabinoids Induce Autophagy and Mitochondrial Apoptotic Pathways in Human Glioblastoma Cells Independently of Deficiency in *TP53* or *PTEN* Tumor Suppressors

**DOI:** 10.3390/cancers13030419

**Published:** 2021-01-22

**Authors:** Aleksandra Ellert-Miklaszewska, Iwona Anna Ciechomska, Bozena Kaminska

**Affiliations:** 1 Laboratory of Molecular Neurobiology, Nencki Institute of Experimental Biology, Polish Academy of Sciences, 02-093 Warsaw, Poland; i.ciechomska@nencki.edu.pl; 2 Affiliated Cancer Hospital & Institute of Guangzhou Medical University, Guangzhou 510095, China

**Keywords:** cannabinoids, glioblastoma, apoptosis, autophagy, mitochondrial apoptotic pathway, mTOR, TP53, PTEN

## Abstract

**Simple Summary:**

Glioblastomas (GBMs) are aggressive brain tumors with frequent genetic defects in *TP53* and *PTEN* tumor suppressor genes, which render tumors refractory to standard chemotherapeutics. Natural and synthetic cannabinoids showed antitumor activity in glioma cells and animal glioma models. Due to differences in the expression of cannabinoid type 2 receptors (CB2), which are abundant in GBMs but absent from a healthy brain, we tested synthetic cannabinoids for their ability to kill numerous glioma cells. We performed multiple biochemical analyses to determine which cell death pathways are activated in human glioma cells. We demonstrate high susceptibility of human glioblastoma cells to synthetic cannabinoids, despite genetic defects contributing to apoptosis resistance, which makes cannabinoids promising anti-glioma therapeutics.

**Abstract:**

Glioblastomas (GBMs) are aggressive brain tumors with frequent genetic alterations in *TP53* and *PTEN* tumor suppressor genes rendering resistance to standard chemotherapeutics. Cannabinoid type 1 and 2 (CB1/CB2) receptor expression in GBMs and antitumor activity of cannabinoids in glioma cells and animal models, raised promises for a targeted treatment of these tumors. The susceptibility of human glioma cells to CB2-agonists and their mechanism of action are not fully elucidated. We determined CB1 and CB2 expression in 14 low-grade and 21 high-grade tumor biopsies, GBM-derived primary cultures and established cell lines. The non-selective CB receptor agonist WIN55,212-2 (but not its inactive enantiomer) or the CB2-selective agonist JWH133 induced apoptosis in patient-derived glioma cultures and five established glioma cell lines despite p53 and/or PTEN deficiency. Growth inhibitory efficacy of cannabinoids correlated with CB1/CB2 expression (EC_50_ WIN55,212-2: 7.36–15.70 µM, JWH133: 12.15–143.20 µM). Treatment with WIN55,212-2 or JWH133 led to activation of the apoptotic mitochondrial pathway and DNA fragmentation. Synthetic cannabinoid action was associated with the induction of autophagy and knockdown of autophagy genes augmented cannabinoid-induced apoptotic cell death. The high susceptibility of human glioblastoma cells to synthetic cannabinoids, despite genetic defects contributing to apoptosis resistance, makes cannabinoids promising anti-glioma therapeutics.

## 1. Introduction

Malignant gliomas are the most common primary neoplasms of the central nervous system (CNS). Glioblastoma (WHO grade IV glioma, GBM) is highly resistant to standard therapeutic approaches, i.e., surgery followed by radiation and chemotherapy [1,2,3]. Despite many attempts to develop more effective clinical treatments against GBMs, a median survival of GBM patients is 15 months from a diagnosis due to invariable recurrence of the disease [4]. Many mechanisms contribute to high drug resistance of human glioblastoma cells, including the presence of glioma stem cells, alterations in the cell cycle inhibitors, and defects in the apoptotic machinery [5,6,7,8,9,10]. Most frequent genetic alterations involve genes coding for tumor suppressors p53 regulating cell cycle and apoptosis [11] and PTEN (phosphatase and tensin homolog deleted on chromosome ten), a lipid phosphatase controlling growth factors induced PI-3K signaling [8,9,12,13]. Mechanistic studies demonstrated that antitumor chemotherapy mediated by p53 succeeds only in tumor cells with a functional PTEN and is lost in PTEN-null cells [14]. Thus, sensitivity to various cytotoxic drugs or radiotherapy may be determined by a status of *TP53* and *PTEN* genes in tumor cells.

The exploitation of natural and synthetic cannabinoids as antitumor compounds has emerged as an attractive topic [15] due to several findings showing their cytotoxic potential against many cancer cells and antitumor activity in animal cancer models, including malignant gliomas [16,17]. Sánchez and co-workers showed that (-)-*trans*-∆^9^-tetrahydrocannabinol (∆^9^-THC) inhibits growth of C6 glioma cells in vitro and induces cell death with features typical for apoptosis [18]. More importantly, the antitumor action of cannabinoids, mediated via the CB1 and/or CB2 cannabinoid receptors, resulted in a significant regression of rat and human malignant gliomas in drug-treated animals [19,20,21,22]. These results prompted the clinical trials of ∆^9^-THC in patients with recurrent GBM [23,24]. Mechanistically, pro-apoptotic and tumor growth-inhibiting activities of cannabinoids were mediated by the accumulation of de novo-synthesized ceramide, which led to cannabinoid-triggered endoplasmic reticulum (ER) stress [21,25,26] and ER-stress-related stimulation of the p8/TRB3 pathway resulting in the induction of autophagy [27]. Autophagy is an evolutionarily conserved catabolic process of degradation of proteins, organelles, and recycling materials in response to cellular stress. The events are accompanied by progressive development of vesicular structures from autophagosomes to autolysosomes [28,29]. Most chemotherapeutics induce cell damage that triggers autophagy, which could be either protective or detrimental depending on the strength of stimuli and cellular context [29,30,31]. ER stress/autophagy cascade, which was triggered by ∆^9^-THC treatment led to apoptotic death of glioma cells [27]. In other cells, for example breast cancer cells, cannabidiol treatment increased apoptosis levels, when autophagy was inhibited [32].

The agonists of CB1 receptor induce psychodysleptic side effects due to widespread expression of this receptor in CNS, therefore selective CB2 receptor agonists are of interest for brain tumor therapy. While CB2 expression is not detectable in non-transformed astrocytes, CB2 has been detected by us and others in some astrocytic tumors and the extent of receptor expression correlates with malignancy grade [22,33,34]. Some studies showed low expression of CB2 in gliomas, which corresponded to the lack of pro-apoptotic effect in cultured glioma cells in response to cannabinoids. Due to some controversies in the field we aimed to provide a systematic evaluation of cannabinoid receptor expression in gliomas and cell lines, as well as characterization of cannabinoids effects on human glioma cells. In the present work, we evaluated the expression of the CB1 and CB2 receptors in tumor biopsies, established human glioma cell lines and in GBM-derived primary cultures. We tested the efficacy of a non-selective CB receptor agonist WIN55,212-2 and a CB2-selective agonist JWH133 towards tumor cells with distinct patterns of CB receptor expression. Given high frequency of *TP53* and *PTEN* defects in gliomas, we studied whether the deficiency of these tumor suppressors restrains antitumor activity of the synthetic cannabinoids. Our results show that both cannabinoids induce apoptosis in human glioma cells. We observed that the appearance of several autophagy features after cannabinoid treatment is preceded by the inhibition of mTOR signaling in glioma cells. Suppression of autophagy by the silencing of essential autophagy genes augmented apoptotic effects of cannabinoids. Altogether, we show the involvement of autophagy pathways into cannabinoid-induced death of malignant glioma cells and present an evidence that autophagy plays cytoprotective rather than cytotoxic role in the process.

## 2. Results

### 2.1. Human Glioblastoma Cells Express CB1 and CB2 Receptors

**Figure 1:**
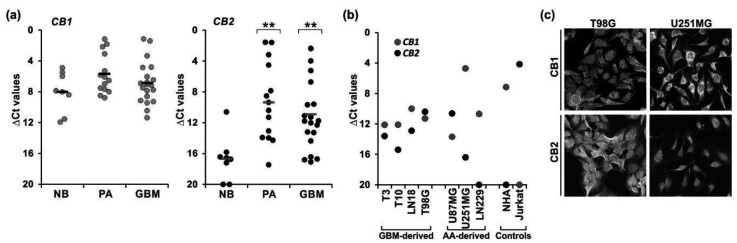
Expression of cannabinoid receptors type 1 (CB1) and 2 (CB2) in tumor samples, and tumor-derived and established human glioblastoma cell cultures. The levels of *CB1* and *CB2* mRNA were analyzed by quantitative RT-PCR (**a**) in tumor biopsies from benign juvenile pilocytic astrocytomas (PA, WHO grade I, *n* = 14) and highly malignant glioblastomas (GBM, WHO grade IV, *n* = 21), as well as in normal human brain samples (NB, *n* = 8, two of the RNA samples being pooled from multiple donors); and (**b**) in human glioblastoma primary cultures: T3 and T10, and established cell lines: T98G, U251MG, U87MG, LN229; GBM—glioblastoma multiforme-derived; AA—anaplastic astrocytoma-derived cell line; normal human astrocytes (NHA) and Jurkat cells (human T-cell lymphoblastic leukemia cells). Results are presented as ∆Ct values (Ct of a target gene—Ct of a reference *GAPDH* gene). For tumor biopsies each individual sample is plotted and a mean in each group is marked with a horizontal line; for cell lines the values correspond to means from two independent preparations in duplicate. (**c**) Representative micrographs showing differential CB1 and CB2 expression in T98G and U251MG cells. The presence of CB1 and CB2 receptors was studied at the protein level by immunocytochemistry and confocal microscopy; original magnification 200×. Statistical significance of differences in gene expression in PA and GBM samples as compared to NB was determined using ANOVA and is indicated as follows: ** *p* < 0.01.

The CB1 and CB2 receptor expression in tumor vs. non-transformed brain tissues was evaluated using the quantitative RT-PCR in benign juvenile pilocytic astrocytomas (PA, WHO grade I, *n* = 14), glioblastomas (GBM, WHO grade IV, *n* = 21), and normal human brain samples (NB, *n* = 8, two of the RNA samples being pooled from multiple donors) (Figure 1a). We also determined their expression in normal human astrocytes (NHA), primary cultures of human GBM cells, and established glioma cell lines (derived from GBMs and WHO grade III astrocytomas—AA) (Figure 1b). The levels of *CB1* mRNAs did not differ between NB, PA, and GBM samples. *CB1* transcript was detected in all examined cell lines but the levels of receptor expression in the majority of glioma cells (except U251MG cells) were lower than those found in NHA. In contrary, *CB2* expression was substantially higher in tumor tissues and cells vs. normal brains and NHA, respectively. Elevated *CB2* levels were observed in both PA and GBM tumor samples. Among the cell lines, the highest *CB2* expression was found in GBM-derived cells (including tumor-derived primary cultures), while *CB2* mRNA was low or undetectable in two out of three cell lines originated from AA, i.e., U251MG and LN229, respectively.

The upregulated levels of *CB2* mRNAs in tumor samples are in line with the increased CB2 immunoreactivity determined in brain tumor sections in our previous study [34]. The profile of cannabinoid receptor expression in glioma cells was confirmed at the protein level using immunocytochemistry. The confocal microscopy examination demonstrated a high level of CB1 expression in U251MG cells and a strong CB2 immuno-reactivity in T98G cells (Figure 1c). There was no CB2-imunoreactivity in LN229 cells.

### 2.2. Cannabinoids Induce Growth Arrest and Cell Death in p53mut and/or PTENmut Human Glioblastoma Cells

The expression of CB1 and CB2 in human glioblastoma cell lines and tumor-derived cultures prompted us to investigate whether these cells would respond to cannabinoids and to what extent susceptibility to cannabinoids would be affected by *TP53* and *PTEN* defects.

**Figure 2:**
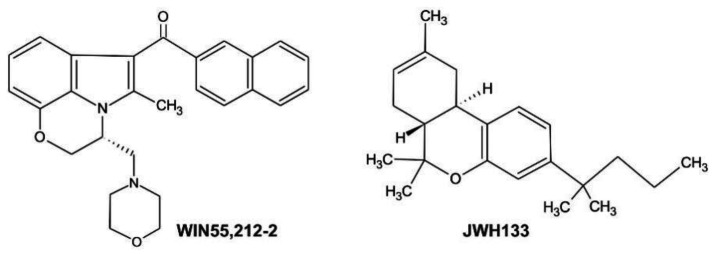
Chemical structures of synthetic cannabinoids used in the study: WIN55,212-2, a CB1/CB2 receptor agonist ((R)-(+)-(2,3-Dihydro-5-methyl-3-(4-morpholinylmethyl)pyrrolo(1,2,3-de)-1,4-benzoxazin-6-yl)-1-naphthalenylmethanone) and JWH133, a selective CB2 receptor agonist ((6aR,10aR)-3-(1,1-Dimethylbutyl)-6a,7,10,10a-tetrahydro-6,6,9-trimethyl-6H-dibenzo[b,d]pyran).

**Figure 3:**
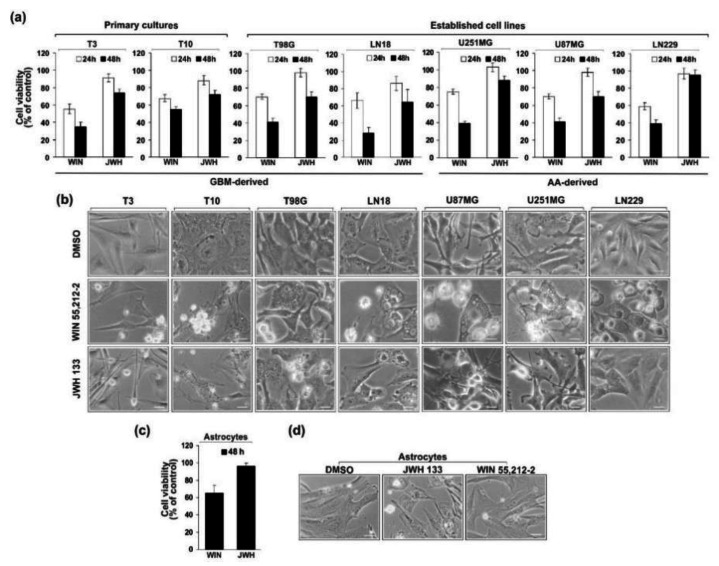
Effect of synthetic cannabinoids WIN55,212-2 or JWH133 on the viability and morphology of glioblastoma cells (**a**,**b**) and normal human astrocytes (**c**,**d**). (**a**,**c**) Cell viability was determined 24 h and 48 h after exposure to cannabinoids using MTT metabolism test. Results are expressed in values relative to DMSO-treated control cells, as the mean ± SEM of at least three independent experiments (each in triplicate). (**b**,**d**) Phase-contrast micrographs show control glioblastoma cells and normal human astrocytes (treated with 0.1% DMSO) with a flattened appearance and typical changes in cellular morphology after 36 h—exposure to 15 µM WIN55,212-2 or 10 µM JWH133. Note the retraction of cell extensions, cell shrinkage, cell membrane blebbing, and the formation of apoptotic bodies upon exposure to cannabinoids, as well as extensive cytoplasm vacuolization in WIN55,212-2-treated cells. Scale bars correspond to 40 µm in T3, T10 cells, and astrocytes, and to 20 µm in established glioma cell lines.

The human glioblastoma cells were treated with 15 µM WIN55,212-2 (CB1/CB2 agonist) or its inactive enantiomer, or 10 µM JWH133 (CB2-selective agonist) (Figure 2). At these concentrations both compounds reduced the viability of rat C6 glioma cells by 50% (EC_50_s at 48 h) [35]. As evidenced by MTT metabolism test, WIN55,212-2 decreased the number of viable cells in all examined cell lines in a time-dependent manner (Figure 3a). The effectiveness of JWH133 towards human glioma cells was lower than the efficacy of WIN55,212-2 at the same time after treatment. The CB2-selective agonist had no influence on LN229 cells survival and only mildly affected the viability of U251MG cells. Both cannabinoids inhibited growth of primary GBM cultures. The response to the synthetic cannabinoids, as reflected by EC_50_s at 48 h (Table 1), corresponded well with the expression of cannabinoid receptors. Decrease in cell viability was accompanied by profound changes in cell morphology as shown in Figure 3b. Upon the same treatment as applied to induce death of human glioma cells, the survival rate of normal human astrocytes was substantially reduced after 48 h to treatment with WIN55,212-2, however it was not affected by JWH133 (Figure 3c). Even a prolonged treatment with the CB2-selective agonist did not decrease NHA viability or produce changes in the cell morphology.

The established glioblastoma cell lines used in our study have been characterized with respect to *TP53* and *PTEN* status as specified in Table 2 [7,12], except that there were inconsistent data concerning p53 activity in LN229 cells [36,37]. We evaluated p53 activities in primary glioma cultures and established glioma cell lines by transfecting cells with plasmids carrying the luciferase reporter gene under the control of a p53-responsive (pPG13-Luc) or mutated promoter (pMG13-Luc, with mutated p53 binding sites).

**Figure 4:**
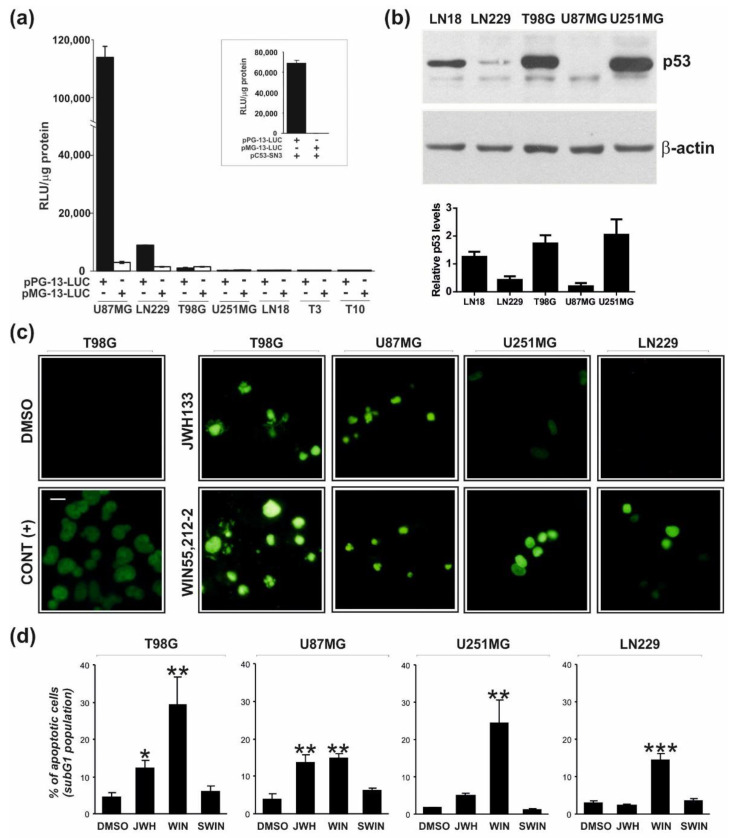
Endogenous p53 transcriptional activity in glioblastoma cells and detection of cannabinoid-induced apoptotic DNA fragmentation by TUNEL and flow cytometry. (**a**) Endogenous p53 transcriptional activity was measured in various glioma cells 24 h after transfection with a reporter plasmid encoding firefly luciferase under a wild type p53-responsive promoter (pPG13-Luc) or a mutated p53 promoter (pMG13-Luc, negative control). Relative luciferase activity is shown as raw light units (RLU) in cell lysates/1 µg of protein. All measurements were done in quadruplicates and the results of a representative experiment (of three independent experiments) are presented as mean ± SEM. A high level of p53-dependent transcriptional activity was observed only in U87MG and LN299 cells. *Inset:* Overexpression of wild-type p53 (pC53-SN3) in p53 null cells, U251MG, restored the ability to activate a p53-responsive promoter. (**b**) A representative immunoblot showing accumulation of p53 in LN18, T98G, and U251MG glioma cells. The graph below shows the levels of p53 normalized to β-actin based on the densitometry analysis (the mean ± SD of at least two independent experiments). (**c**,**d**) Apoptotic DNA-fragmentation was evaluated in glioblastoma cells after 48 h-treatment with WIN55,212-2, its inactive enantiomer WIN55,212-3 (SWIN), JWH133, or DMSO (vehicle). (**c**) DNA strand breaks were detected in situ using fluorescein-labeled nucleotides (green fluorescence) in TUNEL reaction as described in Materials and Methods. DNase treated cells were used as a positive control (CONT (+)). A scale bar corresponds to 20 µm. (**d**) DNA content was measured by flow cytometry in propidium iodide stained cells. Bars on the graph show the average (±SEM) percentage of apoptotic cells in the subG1 cell cycle phase determined from histograms analysis in three independent experiments. Statistical significance of changes after exposure to WIN55,212-2 or JWH133 as compared to control cells (DMSO) was determined using ANOVA and is indicated as follows: * *p* < 0.05, ** *p* < 0.01, *** *p* < 0.001.

Transfection itself was a sufficient stimulus to transiently induce an endogenous p53 transcriptional activity. The p53-responsive promoter was activated in the U87MG and LN229 cells, that suggests the presence of functional p53 (Figure 4a). No endogenous p53 activity was detected in primary T3 and T10 cell cultures, U251MG, and T98G cells. Up-regulation of the p53-responsive (but not mutated) promoter activity upon overexpression of the wild type p53 in U251MG cells confirmed the sensitivity and specificity of the assay (Figure 4a, *Inset*). We observed increased p53 protein levels and the nuclear accumulation of p53, respectively in LN18, T98G, and U251MG cells using immunoblotting (Figure 4b) and cell immunostaining (Figure S1). These results are in agreement with the analysis of p53 transcriptional activity as a wild-type p53 is rapidly degraded and mutant forms are stabilized in tumor cells [38].

We found reduced levels of phosphorylated retinoblastoma (Rb) protein, a main regulator of the cell cycle [39] in cells treated with WIN55,212-2 and in CB2-expressing cells treated with JWH133. Rb protein is a tumor suppressor that in non-phosphorylated state arrests cell proliferation [40].

The cannabinoid treatment triggered apoptotic DNA fragmentation as evidenced by an increase in the number of TUNEL-positive cells in responsive glioma cells (Figure 4c,). Furthermore, we quantified the percentages of cells with fragmented DNA (an apoptotic subG1 population) using flow cytometry. As shown in Figure 4d, WIN55,212-2, but not its inactive enantiomer WIN55,212-3 or DMSO, induced a statistically significant increase in the number of apoptotic cells after 48 h treatment in all tested cell lines. T98G and U87MG cells were also sensitive to JWH133, while U251MG and LN229 were not susceptible to treatment with the CB2-selective drug. The increase of the apoptotic population in U251MG cells after JWH133 treatment was not statistically significant. The subG1 population in cannabinoid-treated U87MG cells appeared with a 6–8 h delay, in comparison to other cell lines.

Thus, our results demonstrate that the expression of CB1 and/or CB2 receptors predispose glioma cells to cannabinoid-induced cell death, while *TP53* and/or *PTEN* defects did not abrogate the pro-apoptotic responses induced by the treatment.

### 2.3. Cannabinoids Trigger Mitochondrial Pathway of Cell Death and Caspase Activation

**Figure 5:**
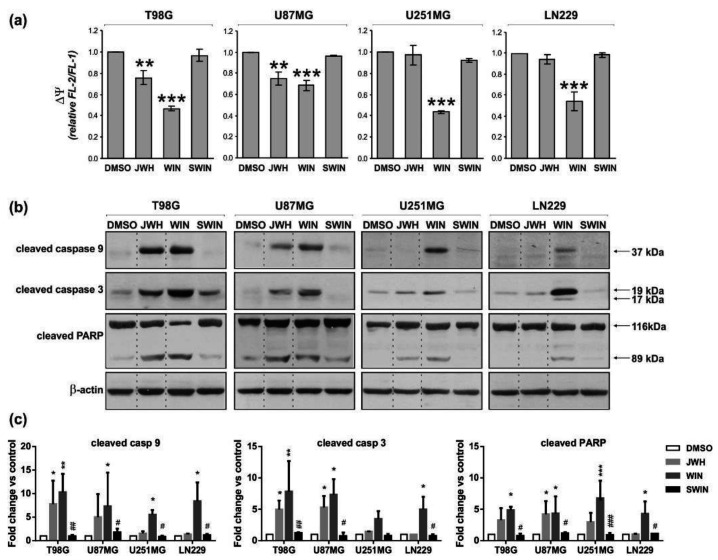
Synthetic cannabinoids induce the dissipation of mitochondrial membrane potential and the activation of apoptotic caspases in glioblastoma cells. (**a**) Flow cytometric analysis of mitochondrial transmembrane potential (∆*Ψ*) was performed in control and cannabinoid-treated cells stained with the fluorescent probe JC-1. A decrease of the red (FL-2 channel)/green (FL-1 channel) fluorescence intensity ratio, normalized to the values from untreated controls, corresponds to a loss of mitochondrial membrane potential. Statistical significance of changes 24 h after exposure to WIN55,212-2 or JWH133 as compared to control cells (DMSO) was determined using ANOVA and is indicated as follows: ** *p* < 0.01, *** *p* < 0.001. The inactive enantiomer WIN55,212-3 (SWIN) did not induce changes in mitochondrial membrane potential in any cell line. (**b**) Synthetic cannabinoids-induced activation of caspase 9 and caspase 3, as indicated by the immunodetection of cleaved proteins, was evaluated at 36 h post-treatment. Caspase 3 activation was also evidenced by PARP proteolysis at 48 h post-treatment. Equal loading of proteins was ensured by β-actin immunodetection. (**c**) The graphs show the relative intensity of bands on immunoblots as compared to the control (DMSO-treated cells) and normalized to β-actin levels based on the densitometry analysis (the mean ± SD of at least two independent experiments). Statistical significance of changes was determined using two-way ANOVA and is indicated as follows: * *p* < 0.05, ** *p* < 0.01, *** *p* < 0.001 (vs. DMSO) and ^#^ *p* < 0.05, ^##^ *p* < 0.01, ^###^ *p* < 0.001 (SWIN vs. WIN).

We have previously reported that apoptosis induced by WIN55,212-2 in rat C6 glioma cells was accompanied by the dissipation of mitochondrial membrane potential [35]. To evaluate an involvement of the mitochondrial apoptotic pathway in the cannabinoid-induced death of human glioblastoma cells, we determined changes in the mitochondrial membrane potential (∆*Ψm*) by flow cytometry using JC-1 staining. Figure 5a shows that WIN55,212-2 (but not its inactive enantiomer WIN55,212-3) decreased the ∆*Ψm* in all tested cells, as compared to DMSO-treated controls. JWH133 induced less pronounced changes than WIN55,212-2 in T98G and U87MG cells, and showed no effect in U251MG and LN299 cells.

The decrease of ∆*Ψm* correlated with the appearance of active caspase 9 and activation of the executory caspase 3. Immunoblot analysis (Figure 5b,c) showed increased levels of cleaved caspases in all tested cells exposed to WIN55,212-2 (its enantiomer WIN55,212-3 was not effective). The significant up-regulation of the active caspase 9 and the active caspase 3 levels was observed in response to JWH133 administration in T98G and U87MG cells. The levels of active caspase 9 and caspase 3 were barely induced in JWH133-treated U251MG cells and the increase was not statistically significant. An apparent lack of caspase 9 activation in JWH133-treated U251MG cells corresponded to the maintenance of ∆*Ψm* at the similar level in these cells. JWH133 treatment did not trigger caspase activation in LN229 cells. The activation of caspase 3 in the cell lines sensitive to cannabinoids was additionally evidenced by the cleavage of its substrate, poly-ADP ribose polymerase (PARP, a DNA-repair enzyme). The levels of active caspases and cleaved PARP in the cells treated with inactive enantiomer WIN55,212-3 were undetectable or as low as in controls. The response to cannabinoids developed gradually over time of treatment as shown for T98G cells (Figure S2).

The changes in mitochondrial membrane potential upon cannabinoid treatment and subsequent activation of the caspase cascade, followed by apoptotic DNA fragmentation confirm the contribution of the mitochondrial apoptotic pathway to WIN55,212-2 and JWH133-induced cell death of human glioma cells.

### 2.4. Cannabinoids Induce Autophagy Associated with Inhibition of mTOR Pathway in Glioblastoma Cells

The profound changes of cell morphology observed upon the treatment prompted us to examine whether the synthetic cannabinoids induce autophagy in glioma cells. First, we evaluated the formation of acidic vesicular organelles (AVOs), associated with autophagy. We found an increase of AVO’s both in T98G and LN18 glioma cells treated with WIN55,212-2 and to a lesser extent with JWH133. As we reported previously [31], T98G cells are characterized by high level of endogenous autophagy, and there are numerous AVO’s detected even in the cells under control conditions (DMSO and S-WIN55,212-3-treatment). The response of U87MG and LN229 cells to WIN55,212-2 was much weaker than of the other cell lines and the effect of JWH133 was barely detectable (Figure S3). More detailed studies on autophagy were carried out on LN18 cells, which express both cannabinoid receptors, show low basal levels of autophagy and an apparent induction of this process upon cannabinoid treatment.

**Figure 6:**
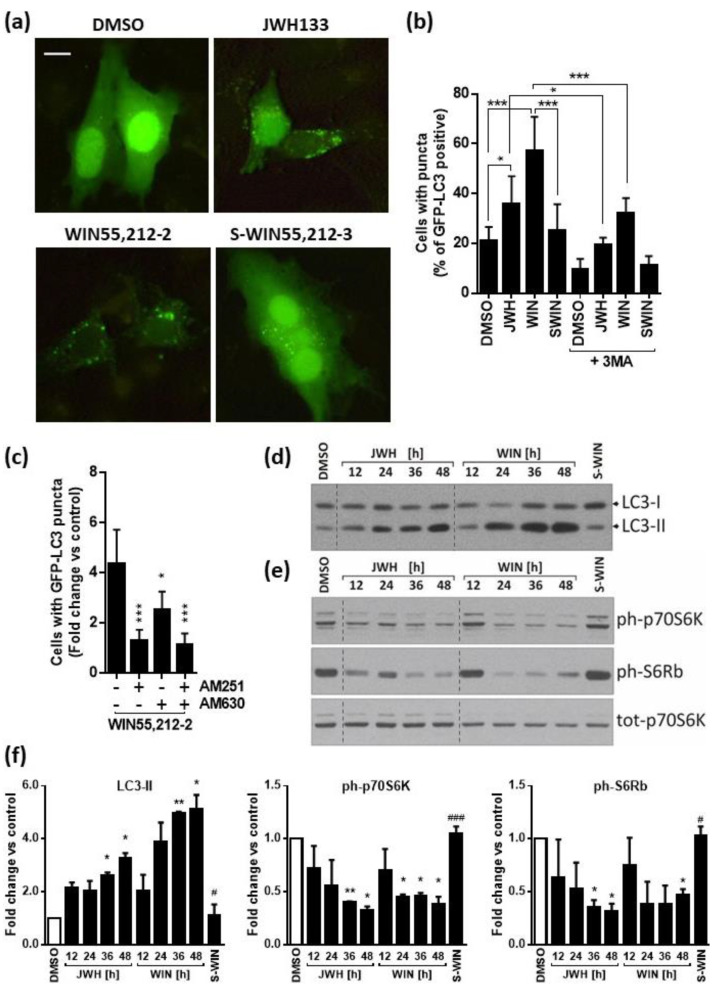
Synthetic cannabinoids induce autophagy associated with inhibition of mTOR pathway. (**a**) Representative microphotographs of LN18 cells transfected with GFP-LC3 and treated with DMSO (vehicle), JWH133, WIN55,212-2 or its inactive enantiomer WIN55,212-3 (SWIN), JWH133 for 24 h show the increase of LC3 punctation in cannabinoid-treated cells. Increase of GFP-LC3–positive cells with GFP-LC3 dots in cells exposed to JWH133 or WIN55,212-2 was reduced following 3-methyladenine (3MA, 2 mM for 24 h). A scale bar corresponds to 10 µm (**b**) and by pre-treatment with antagonists of CB1 receptor (AM251) and/or CB2 receptor (AM630) in WIN55,212-2-treated cells. Statistical significance of changes was determined using one-way ANOVA and is indicated as follows: * *p* < 0.05, *** *p* < 0.001. (**c**). Cells were scored for the presence of GFP-LC3 puncta (more than five dots per cell) among GFP-LC3-transfected cells. A minimum of 100 cells per sample were counted (mean ± SEM, three independent experiments). One-way ANOVA followed by Fisher post-hoc test; * *p* < 0.05, *** *p* < 0.001. (**d**,**e**) LN18 glioma cells were exposed to synthetic cannabinoids JWH133 (JWH) or WIN55,212-2 (WIN) and control treatments with DMSO or WIN-55,212-3 (S-WIN) for 12–48 h. Representative immunoblots show the effects on LC3-I and LC3-II levels (**d**) and on the phosphorylation level of mTORC1 downstream substrate: p70 S6 kinase and its target S6 ribosomal protein (**e**). (**f**) The graphs show the relative intensity of the bands on immunoblots (LC3-II, ph-p70S6K, ph-S6Rb) as compared to the control (DMSO-treated cells) and normalized to total p70S6K levels; based on the densitometric evaluation (the mean ± SD of at least two independent experiments). Statistical significance of changes was determined using one-way ANOVA and is indicated as follows: * *p* < 0.05, ** *p* < 0.01 (vs. DMSO) and ^#^ *p* < 0.05, ^###^ *p* < 0.001 (SWIN vs. WIN).

During the initiation step of autophagy, lipid conjugation of microtubule-associated protein light chain 3 (LC3) leads to the conversion of the soluble form of LC3 (LC3-I) to the autophagic-vesicle-associated form (LC3-II). The GFP-tagged LC3 plasmid was used as a specific tool to detect autophagy in cultured glioma cells. As shown in Figure 6a, the distribution of GFP-LC3 in transfected untreated cells was diffused, with a minority of cells presenting GFP-LC3 dots. The treatment with WIN55,212-2 or JWH133 for 24 h increased GFP-LC3 punctation and the percentage of cells showing GFP-LC3 dots was higher upon exposure to CB1/CB2 agonist than to CB2-specific cannabinoid. The increase of GFP-LC3 punctation in glioma cells upon the treatment with both cannabinoids was statistically significant and the induced changes were partially blocked by the autophagy inhibitor 3-methyladenine (3MA) (Figure 6b). Moreover, a number of cells with punctate GFP-LC3 pattern was reduced by pre-treatment of cells with antagonists of CB1 receptor (AM251) and/or CB2 receptor (AM630) before the exposure to WIN55,212-2 (Figure 6c). Immunoblots demonstrate the time-dependent accumulation of endogenous LC3-II in glioma cells treated with JWH133 and even more pronounced changes induced by WIN55,212-2 at the same time points (Figure 6d).

Mammalian target of rapamycin (mTOR) Complex 1 (mTORC1) is a known suppressor of autophagy. We found that the levels of phosphorylated 70 kDa ribosomal S6 kinase (p70S6K), the direct downstream effector of mTORC1, and the levels of phosphorylated S6 ribosomal protein (pS6Rb), a p70S6K substrate, decreased gradually in LN18 glioma cells after the exposure to cannabinoids (Figure 6e,f). Altogether, these results demonstrate that WIN55,212-2 and JWH133 induce autophagy in LN18 glioma cells, which was associated with the inhibition of mTORC1.

The responses of other glioma cells, i.e., T98G, U87MG, and LN229, are presented in Figure S4. WIN55,212-2 treatment induced the activation of autophagy in T98G and LN229 cells as evidenced by the accumulation of LC3-II (Figure S4a). A co-incubation with BafA1, which blocks lysosomal degradation of LC3-II, enhanced the cannabinoid-induced accumulation of LC3-II, suggesting dynamic autophagy in glioma cells upon the cannabinoid treatment. There was no increase of LC3-II upon JWH133 treatment as compared to the control levels. The levels of phosphorylated p70S6K and pS6Rb were decreased in WIN55,212-2 treated T98G and LN229 cells, pointing to the inhibition of mTOR pathway (Figure S4b). We did not detect an induction of autophagy in U87MG cells under these treatment conditions.

### 2.5. Inhibition of Autophagy Augments Cannabinoids-Induced Apoptotic Cell Death

**Figure 7:**
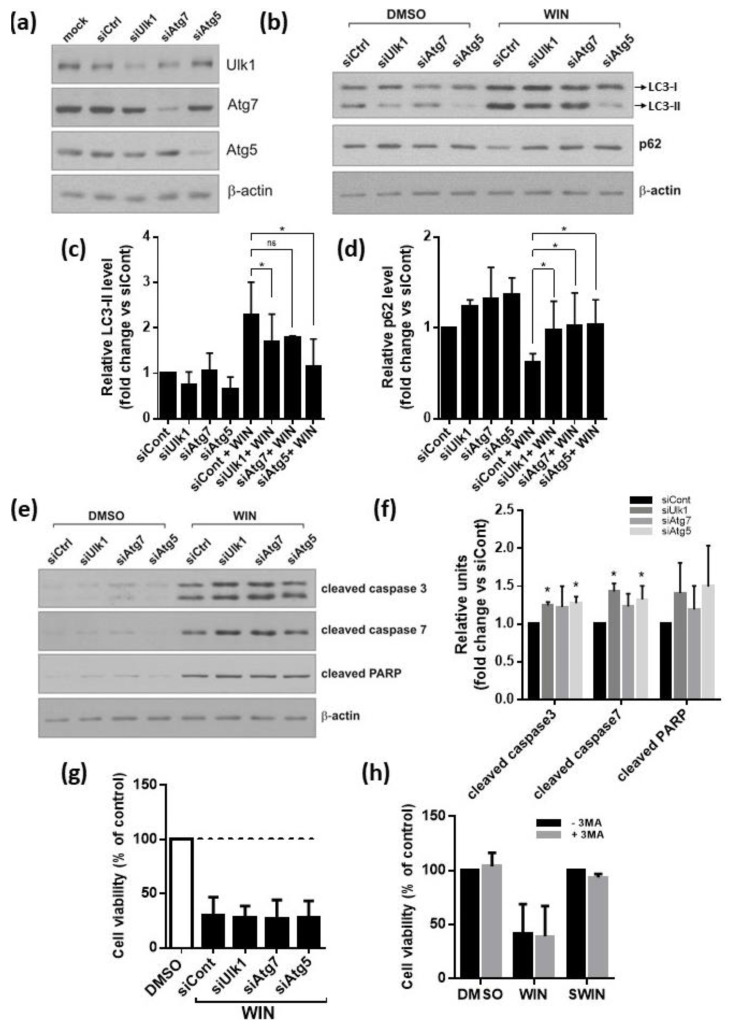
The inhibition of autophagy augments cannabinoid-induced apoptotic cell death in malignant glioma cells but does not affect its overall cytotoxicity. (**a**) Knockdown of ULK1, Atg5, and Atg7 using specific siRNAs was confirmed at protein level by immunoblotting. (**b**–**e**) Glioma cells were transfected with siRNA targeting Atgs and exposed to WIN55,212-2 for 24h. Immunoblots show the levels LC3-I and LC3-II, p62 (**b**), and cleaved caspases 3, 7, and PARP (**e**) in glioma cells. Note the increase of apoptosis hallmarks in WIN55,212-2-treated LN18 glioma cells after inhibition of autophagy. Densitometric analysis of immunoblots presented in (**b**) and (**e**) is shown in (**c**,**d**) and (**f**), respectively. Each bar represents the mean ± SD of three independent experiments. In (**c**,**d**,**f**) significant difference (* *p* < 0.05, ANOVA) as compared to WIN55,212-2-treated siCont-transfected cells; ns—not statistically significant. (**g**) Viability of LN18 glioma cells treated for 48 h with WIN55,212-2 after knockdown of autophagy genes, assessed by MTT metabolism test. Values were normalized to DMSO-treated cells transfected with the respective siRNA. Results shown represent mean ± SD from three independent experiments. No significant differences in cell viability after exposure to WIN55,212-2 between cells transfected with siCont and with siRNA targeting any of the Atg genes were found (ANOVA). (**h**) Viability of LN18 glioma cells measured by MTT metabolism test after 48 h-treatment with DMSO, WIN55,212-2, or WIN55,212-3 (SWIN) and with or without addition of 3-methyladenine (3MA, 2mM). Values were normalized to DMSO-treated cells. Results shown represent mean ± SD from three independent experiments. No significant differences in the response of cells to WIN55,212-2 were found between cells with and without 3MA co-treatment (ANOVA).

Due to its dual nature, the inhibition of different stages of autophagy may result in different outcomes in glioma cells [41,42]. We silenced Atg1/ULK1, implicated in the initiation of autophagy, and Atg5 and Atg7, required for the autophagosome formation. We observed effective silencing of ULK1, Atg5, Atg7 at the protein levels (Figure 7a). The autophagy-related increase of the lipidated LC3-II isoform induced by WIN55,212-2 in LN18 cells was partially abrogated by the knockdown of Atg genes (Figure 7b,c). The strongest inhibitory effect was observed in Atg5 siRNA-transfected cells (Figure 7c). The p62 protein regulates the removal of protein aggregates and damaged organelles and it is itself degraded during autophagy [43]. The decrease in p62 levels observed in WIN55,212-2-treated cells was blocked by Atg genes silencing, confirming efficient inhibition of autophagy flux (Figure 7b,d). WIN55,212-2-induced cleavage of caspases 3, 7, and PARP was augmented in LN18 cells after knockdown of *ULK1*, *Atg5,* or *Atg7* expression (Figure 7e,f). The quantification of cleaved caspases and PARP levels demonstrated that the apoptosis rate in cannabinoid-treated, Atg5, or ULK1 depleted cells was statistically higher than in the control siRNA–transfected cells (siControl). Similar, although weaker effects, were produced by the silencing of *Atg7*. In summary, we found that a genetic inhibition of autophagy enhanced the apoptotic response, suggesting a protective role of autophagy in the cannabinoid-induced cell death. Along this way, pre-treatment of cells with 3MA, the pharmacological inhibitor of autophagy, resulted in the decreased conversion of LC3 and the increased levels of apoptosis markers: Cleaved caspase 3 and PARP. Nonetheless, the overall cytotoxicity of WIN55,212-2 in LN18 cells remained unchanged, when the autophagy was blocked either by silencing of Atg genes or 3MA treatment, as analyzed by MTT metabolism test (Figure 7g,h). The decrease of cell viability induced by WIN55,212-2 in the other tested cell lines was also not affected by 3MA (Figure S4c).

## 3. Discussion

We and others demonstrated the expression of CB1 and CB2 receptors in rat C6 glioma cells [21] and biopsies from human GBMs and some lower grade astrocytomas [22,34,44,45], as well as cytotoxic effects of cannabinoids in certain cancer cells [19,22,35,46,47]. While the expression of CB2 receptors in bulk PA and GBM tumor samples could be explained by their expression in peripheral immune cells infiltrating the tumor, the expression of CB2 receptors in several established human glioblastoma cell lines and GBM primary cultures presented in this study verifies the presence of these receptors in human glioma cells. We show that in contrast to the *CB1* mRNA expression, which was comparable in all studied cells and normal human astrocytes, *CB2* mRNA was detected primarily in cells originated from glioblastomas, while low or undetectable levels of *CB2* mRNA were observed in NHA and some astrocytoma-derived cell lines. More importantly, CB2 expression was elevated in tumor samples, both in PAs and GBMs, as compared with normal human brain samples. We confirmed the presence of CB2 receptor on glioma cells by immunocytochemistry. Our findings are in agreement with previous reports of CB2 immunodetection in a number of astrocytic tumors [22,34]. De Jesus et al. (2010) showed the decreased CB1 receptor expression in tumor samples [44], in contrast to unchanged [48] or increased CB1 receptor expression in glioblastomas as compared to low grade gliomas and non-tumor brain tissue presented by others [45]. We did not detect any significant difference in the *CB1* expression between PA, GBM, and normal brain samples. Although the presence of *CB2* expression in certain rodent brain populations has been reported [33,49,50], we did not detect *CB2* expression in NHA and *CB2* mRNA levels were very low in normal brain samples, including samples pooled from multiple donors.

We found that the level of CB1 and CB2 receptor expression in cultured human glioma cells correlates with the sensitivity to cannabinoid treatment. The induction of apoptotic death was evidenced by the detection of caspase cascade activation, appearance of apoptotic alterations of cell nuclei, and DNA fragmentation in a panel of AA- and GBM-derived cells. WIN55,212-2, a non-selective cannabinoid, was highly effective in all tested cell lines. The effects evoked by WIN55,212-2 were stereoselective, suggesting a receptor-mediated mechanism. WIN55,212-3, a receptor-inactive enantiomer, did not affect cell viability or caspase activation. Action via the CB2 receptor using the selective agonist JWH133 was sufficient to induce apoptosis in the majority of glioblastoma cells and only LN229 cells, lacking CB2 receptor expression, showed no response to this compound. The lack of vulnerability to JWH133 in LN229 cells and NHA, which do not express this type of receptor, suggest that the induction of apoptosis by JWH133 in the responsive cells was receptor-mediated.

In vitro [18] and in vivo [21] studies demonstrated that CB1/CB2-acting cannabinoids, such as ∆^9^-THC and WIN55,212-2, trigger apoptosis in cancer cells, but not in non-transformed neural cells. However, in our studies, WIN55,212-2 was toxic in human astrocytes and glioma cells, as previously shown by McAllister et al. in human primary mixed glial cultures vs. glioblastoma cells [46]. A novel cannabinoid agonist exhibiting over 10-times higher affinity for the CB2 vs. CB1 receptor produced a minimal toxicity to cultured brain slices at micromolar concentrations required to eradicate U87MG glioma cells [19]. We show the lack of JWH133 cytotoxicity towards human normal astrocytes. The negligible expression of CB2 receptor in the brain may confer a relative safety of CB2-selective agonists for therapy of brain tumors, which show high levels of this type of cannabinoid receptor.

Human glioblastomas exhibit various alterations in the regulation of the cell cycle and induction of apoptotic pathways, which may render them refractory to many chemotherapeutic drugs [2,3,5,6,8,12]. Potential therapeutic efficacy of cannabinoid-based treatments would critically depend on their ability to activate a cell death program in glioblastoma cells. Population studies showed that *TP53* mutations were frequently found in low-grade astrocytomas, including gemistocytic astrocytomas (88%), fibrillary astrocytomas (53%), and oligoastrocytomas (44%). *TP53* was mutated in 31% of primary glioblastomas and an incidence increased up to 65% in secondary glioblastomas [4,51]. About 30–44% of high-grade gliomas, particularly primary glioblastomas, show mutations and high incidence of loss of the *PTEN* gene [4]. These alterations in both tumor suppressor genes are strongly associated with tumorigenesis and malignant progression of gliomas, as well as with resistance to conventional anticancer treatments. Identifying drugs that can induce p53 and/or PTEN independent apoptosis is of utmost importance. We found that alterations in the *TP53* and *PTEN* genes do not abolish cannabinoid-induced cell death of glioma cells. More importantly, we demonstrated that the synthetic cannabinoids show a pro-apoptotic efficacy also in the primary cultures of GBM-derived cells (T3 and T10 cell lines), in which we identified a functional p53 deficiency. It is worth mentioning that glioblastoma cell lines used in the study exhibit alterations of *TP53*, *PTEN* genes and deletions of cell cycle suppressors *p16/CDKN2A* and *p14^ARF^* genes [12]. Thus, the synthetic cannabinoids were able to override alterations of growth regulatory and apoptotic pathways, and induce apoptosis in tumor cells resistant to many conventional anti-cancer drugs.

In the present study, we addressed an issue of which mechanism of cell death is responsible for cannabinoid-induced cytotoxicity in glioma cells. We have previously described changes of the mitochondrial membrane potential and the activation of caspase 9 prior to the cleavage of executory caspases in WIN55,212-2-treated rat C6 glioma cells [35]. The involvement of mitochondria and caspases has been shown in ∆^9^-THC-induced apoptosis in U87MG glioma cells [25]. Our current findings demonstrate that synthetic cannabinoids induce apoptosis in a panel of human glioblastoma cells by promoting the collapse of mitochondrial potential. The contribution of the mitochondrial pathway to apoptosis triggered by a selective CB2 receptor agonist has been demonstrated in Jurkat cells [52], but our report is the first to show this mechanism in non-immune system cells. In tested glioblastoma cell lines expressing CB1 and CB2 cannabinoid receptors, both WIN55,212-2 and JWH133 disturbed the mitochondrial membrane potential and induced a subsequent cleavage of caspase 9 leading to the execution of apoptotic cell death using the same mitochondria-dependent pathway.

The pro-apoptotic activity of cannabinoids acting via CB1 and CB2 receptors relies on the accumulation of ceramide [21,26]. Mechanistic studies proposed a mechanism, which links ceramide and the execution of ∆^9^-THC-induced cell death via apoptotic mitochondrial pathway [25,27,53]. According to this mechanism, increased ceramide levels, which accumulate in the ER in the response to ∆^9^-THC treatment, trigger activation of eIF2α by a protein kinase-like endoplasmic reticulum kinase, PERK [27]. This leads to transcriptional up-regulation of the co-activator protein p8 and its downstream targets, such as ATF4, CHOP, and TRB3, and thus to activation of the ER stress response [25]. Next, the increased expression of TRB3 promotes its interaction with Akt and leads to decreased phosphorylation of Akt, as well as of its direct substrates TSC2 (tuberous sclerosis protein 2, tuberin) and PRAS40 (the proline-rich Akt substrate of 40 kilodaltons). This, in turn, results in mTORC1 inhibition and the induction of autophagy followed by apoptotic glioma cell death [27]. Some cannabinoids show their antiproliferative effects in tumor cells independently of cannabinoid-receptor binding and via an alternative mechanism. In glioma cells and breast cancer cells, cannabidiol, a phytocannabinoid with a negligible affinity to CB1 or CB2, increased the generation of reactive oxygen species (ROS), which was linked to a later induction of apoptosis and autophagy [20,32]. Moreover, arachidonoyl cyclopropamide or GW405833, two synthetic cannabinoid ligands specific for CB1 and CB2, respectively, induced ROS-mediated autophagy in pancreatic adenocarcinoma cell lines [54]. We demonstrate that the treatment with synthetic cannabinoids is associated with the concomitant induction of apoptosis and autophagy. The activation of autophagy was evidenced by the increase of acidic vesicular organelles formation and processing of the autophagy marker LC3 to its autophagic-vesicle-associated form (LC3-II). The response of all tested cell lines was higher upon exposure to a CB1/CB2 agonist, WIN55,212-2, than to a CB2-specific cannabinoid, JWH133. Moreover, blocking of CB1 receptor was more effective in the inhibition of WIN55,212-2-induced autophagy than the use of a CB2 antagonist. This suggest that action via CB1 receptor may be crucial to the initiation of autophagy in glioma cells. The use of caspase inhibitors and potent antioxidants, such as α-tocopherol, could enable us to elucidate the mechanism more deeply. However, in this study, we focused on the efficacy of cannabinoids in glioma cells with major genetic defects, namely *PTEN* and *TP53* mutations, and interrelation between autophagy and apoptosis. We observed that the selective knockdown of ULK1, Atg5, or Atg7 or pharmacological inhibition of autophagy with 3MA increased the levels of active caspase 3, 7 and PARP degradation in WIN55,212-2-treated LN18 glioma cells. This shows that blocking autophagy aggravated the process of apoptosis. As we did not observe any significant changes in cell viability, our results suggest that autophagy was initiated rather as a protective mechanism in LN18 cells, in contrast to cell-death promoting effect observed by Salazar et al. in U87MG cells [27]. Autophagy may defend the cell from adverse conditions or contribute to tumor cell death [29]. The mechanisms that switch the impact of autophagy on cell fate and regulate a balance between apoptotic and autophagic cell death are still unclear [55]. We speculate that the difference in the role of cannabinoid-induced autophagy in glioma cells observed in different studies may originate from distinct molecular background of the cells, for example the functional status of p53 (wt*TP53* in U87MG and mut*TP53* in LN18 cells). In other types of cancer cells, derived from melanoma, hepatocellular carcinoma, or breast cancer, cannabinoid-induced autophagy once co-occurring with apoptosis was cytotoxic in cells known to have wild-type *TP53* [56,57] and played a cytoprotective role in MDA-MB-231 cells [32], which have defects in *TP53.* Experimental evidence supports an idea that p53 can act as either an activator or an inhibitor of both autophagy and apoptosis depending on its subcellular localization and this mode of action is beyond its transactivation function. Our finding is of particular significance as mutant variants of p53 accumulate in the tumor cells, which may affect the balance between these two processes [58].

## 4. Materials and Methods

### 4.1. Materials

WIN55,212-2 and WIN55,212-3 were from Sigma Aldrich (Munich, Germany). JWH133, AM251, and AM630 were from Tocris Bioscience. Antibodies used for Western blotting were from Cell Signaling Technology (Beverly, MA, USA) except from anti-LC3 and anti-β-actin-peroxidase conjugated antibody (Sigma-Aldrich, Munich, Germany) and anti-ULK1 antibody (Santa Cruz Biotechnology, Dallas, TX, USA). Antibodies and detection reagents used for immunocytochemistry are listed in method descriptions. JC-1 (5,5′,6,6′-tetrachloro-1,1′,3,3′-tetraethylbenzimidazolecarbocyanine iodide) and propidium iodide were purchased from Molecular Probes. Lipofectamine 2000 was from Invitrogen (Thermo Fisher Scientific, Waltham, MA, USA). Nitrocellulose membrane and enhanced chemiluminescence detection system (ECL) were from Amersham Pharmacia Biotech (Rockford, IL USA). MTT (3-(4,5-dimethylthiazol-2-yl)-2,5-diphenyltetrazolium bromide) and all other reagents were purchased from Sigma Aldrich (Munich, Germany).

### 4.2. Cell Culture and Treatment

The established human glioblastoma cell lines: T98G and LN18 (derived from a glioblastoma multiforme, WHO grade IV), LN229, U251MG, and U87MG (derived from astrocytomas WHO grade III) were from ATCC. Primary human glioblastoma cell lines: T3 and T10 were a kind gift from Dr. Helmut Kettenmann, Max Delbruck Center, Berlin. Cells were grown in Dulbecco’s Modified Eagle’s Medium supplemented with 10% FBS (fetal bovine serum, Gibco, Life Technologies, Rockville, MD, USA) and antibiotics (50 U/mL penicillin, 50 µg/mL streptomycin) under standard conditions. Before the experiment, the cells were transferred for 24 h to 1% FCS-supplemented medium. Cells were treated with JWH133, WIN55,212-2, or its inactive enantiomer WIN55,212-3, or corresponding doses of the solvent (DMSO, max. concentration 0.1%). Bafilomycin A1 (BafA1, 10 nM) and 3-methyladenine (3MA, 2 mM) were used as inhibitors of autophagy. CB1 receptor antagonist (AM251, 5 µM) and CB2 receptor antagonist/inverse agonist (AM630, 5 µM) were added to cell cultures 1 h prior to cannabinoid treatment. The effects of the compounds were monitored at various times by phase-contrast microscopy.

### 4.3. Tissue Samples

Frozen glioma biopsies were obtained from the Brain Tumour Tissue Bank (London Health Sciences Centre, London, ON, Canada). Additional pilocytic astrocytoma specimens were obtained under protocol no. 14/KBE/2012, approved by the Committee of Bioethics at the Children’s Memorial Health Institute (Warsaw, Poland). Informed consent was obtained from all subjects. The reference human adult normal brain RNA samples were from BioChain Institute Inc. (Newark, CA, USA), Clontech Takara (human cerebral cortex total RNA, pooled from 5 male brains, Mountain View, CA, USA) and Ambion (Ambion FirstChoice Human Brain Reference RNA, Thermo Fisher Scientific, Waltham, MA, USA, pooled from 23 normal male/female brains).

### 4.4. Cell Viability—MTT Metabolism Assay

Cells were cultured in 96-well plates with the addition of cannabinoids or with the solvent alone. Following treatment, MTT stock solution was added to each well to a final concentration of 0.5 mg/mL. After 2 h, formazan crystals that formed from MTT in actively metabolizing cells were dissolved in lysis buffer containing 20% SDS and 50% DMF. Optical densities were measured at 570 nm using a scanning multiwell spectrophotometer. Dose–response graphs (log of molar concentrations vs. percent of viable cells) were generated from triplicate observations and the EC50 values were determined.

### 4.5. Quantitative RT-PCR Analysis

Total RNA was isolated according to manufacturer’s protocol (Qiagen, Hilden, Germany), including a DNase digestion step. cDNAs were synthesized by extension of oligo(dT)_15_ primers with 200 units of M-MLV reverse transcriptase in a mixture containing 1 μg of total RNA in 20 μL. Real-time PCR analysis was performed using the ABI-Prism7700 sequence detection system (Applied Biosystems, Waltham, MA, USA) on cDNA equivalent to 10 ng RNA in 20 μL reaction volume containing 1× SYBR Green PCR master mix (Applied Biosystems, Foster City, CA, USA) and 0.4 μM of each primer. The following primers were used: Human GAPDH sense (5′-ATCACCATCTTCCAGGAGCGA-3′) and antisense (5′-AGCCTTCTCCATGGTGGTGAA-3′); human CB1 sense (5′-GATACCACCTTCCGCACCAT-3′) and antisense (5′-TACCCTAATTTGGATGCCATGTC-3′); human CB2 sense (5′-TCATCTACACCTATGGGCATGTTCT-3′) and antisense (5′-CCTCATTCGGGCCATTCC-3′). The thermal cycling conditions were as follows: 50 °C for 2 min, 95 °C for 10 min, followed by 40 cycles of 15 sec at 95 °C for denaturation and 1 min at 60 °C for annealing and extension. The specificity of the PCR reaction was confirmed by a single peak in the dissociation curve. Each pair of primers was validated for equal amplification efficiency to primers of the endogenous reference (GAPDH) at a wide range of cDNA concentrations. Ct, the threshold cycle, was determined after setting the threshold in the linear amplification phase of the PCR reaction and averaged for each sample assayed in triplicates. ∆Ct for a particular gene was defined as Ct(CB1 or CB2)-Ct(GAPDH). Total RNA from two samples of normal human brain (Ambion, Clotech, Mountain View, CA, USA) and isolated from Jurkat cells served as positive controls.

### 4.6. Immunocytochemistry and Acridine Orange Staining

Cells grown on glass coverslips were fixed in 4% paraformaldehyde for 10 min at room temperature and permeabilized with 0.3% Triton X-100. Immunostaining was performed with rabbit anti-human CB1 (Alexis Biochemicals, Enzo Life Sciences, Inc., Farmingdale, NY, USA) or anti-human CB2 (Cayman Chemicals, Ann Arbor, MI, USA) receptor antibodies. The cells were then incubated with biotinylated secondary antibody (Vector Laboratories, Inc., Burlingame, CA, USA) and subsequently with avidin-FITC (Roche Chemicals, Mannheim, Germany). After final washing in PBS, the coverslips were dried, mounted on slides, and visualized by fluorescence microscopy using excitation at 450–490 nm for FITC.

To detect and quantify the acidic vesicular organelles (AVOs) in cells, the vital staining with acridine orange (1 µg/mL for 15 min) was performed. Red fluorescence emission was visualized by fluorescence microscopy.

### 4.7. Transfection with Plasmid DNA for Detection of Autophagy and p53-Responsive Luciferase Reporter Assay

Transfections were performed using Lipofectamine 2000 reagent (Invitrogen, Thermo Fisher Scientific, Waltham, MA, USA). For detection of autophagy cells were transfected with a plasmid coding for GFP-LC3 (kindly provided by Prof. Aviva Tolkovsky, University of Cambridge, Cambridge, UK) and 24 h after transfection were treated with drugs for indicated period of time, then fixed with 3% PFA. The pattern of fluorescent autophagy marker GFP-LC3 in glioma cells was analyzed using fluorescence microscopy. A minimum of 100 cells per culture well were counted with at least two replicates in each of at least three experiments.

For analysis of p53 transcriptional activity, cells were transfected with 1 µg plasmid DNA/well (0.5 µg of each plasmid). We employed two reporter constructs, kindly provided by Dr. M. Hetman (University of Louisville, Louisville, KY, USA), encoding firefly luciferase under a p53-responsive promoter (pPG13-Luc) containing 13 copies of the p53-binding sequence, or a promoter (pMG13-Luc) containing mutated sequence with no ability to bind p53 [59]. The cells were co-transfected with a plasmid coding for *Renilla* luciferase under a constitutive CMV promoter (pRL-CMV). Alternatively, a construct encoding a wild-type-p53 under the CMV promoter (pC53-SN3) [60] was used in co-transfection experiments to test the sensitivity of the promoters. Twenty-four hours after transfection the cells were lysed in 50 µL of a passive lysis buffer (Promega, Fitchburg, WI, USA) and the luciferase activities were measured using the Dual-Luciferase Reporter System (Promega). Luciferase activities were normalized to the amount of protein in cell lysates, determined by the BCA Protein Assay Kit (Pierce, Thermo Fisher Scientific, Rockford, IL, USA). Cells were transfected in quadruplicates in each of three independent experiments.

### 4.8. Silencing of Autophagy Gene Expression with siRNA

On-TARGET plus SMART pool siRNAs targeting human ULK1, Atg5, and Atg7 from Dharmacon (Thermo Scientific, Lafayette, CO, USA) were delivered to glioma cells by electroporation using Nucleofector 2b system (Lonza, Cologne, Germany). Each On-TARGET plus SMART pool of four targeting siRNAs; Ctr siRNA is a pool of non-targeting siRNAs. After 48 h the cells were treated with cannabinoids for the next 24 h, then harvested and analyzed for cell viability, Western blot. Gene knockdown efficacy was determined by Western blots.

### 4.9. Western Blot Analysis

Protein extracts were prepared from floating and culture plate-attached cells as previously described [35] and resolved on SDS-PAGE before electrophoretic transfer onto a nitrocellulose membrane. After blocking in 5% low-fat milk in TBS-T (0.1% Tween 20/Tris-buffered saline, pH 7.6) the membranes were incubated overnight with primary antibodies diluted in blocking buffer and then for 1-h with relevant secondary antibodies. Immunocomplexes were detected using an enhanced chemiluminescence detection system and membrane exposure to X-ray film. The molecular weight of proteins was estimated with pre-stained protein markers. Densitometric analysis was performed using NIH ImageJ software.

LC3 conversion assay. Lipidated LC3-II migrates more rapidly (~16 kDa) than LC3-I (~18 kDa) when proteins are separated by SDS-PAGE. Intensities of LC3-II band were quantified. The numbers below the blots represent average of duplicate or triplicate densitometric analysis of LC3-II normalized to the DMSO control.

Autophagic flux. To exclude that LC3-II increase is induced by inhibition of lysosomal degradation, treated cells were co-incubated with 10 nM bafilomycin A1 (BafA1) for the last 4 h.

### 4.10. TUNEL Staining

Cells cultured on glass coverslips were fixed in 4% paraformaldehyde and permeabilized in 0.1% sodium citrate in 0.1% Triton X-100 in PBS. DNA strand breaks were detected using fluorescein-labeled nucleotides in TUNEL (Terminal transferase-mediated dUTP Nick End Labeling) reaction mixture, according to manufacturer’s protocol. DN-ase-treated cells were used as a positive control. After final washing in PBS, the coverslips were dried, mounted on slides and visualized by fluorescence microscopy using excitation at 450–490 nm.

### 4.11. Flow Cytometry

Measurement of the DNA content (for evaluation of the cell cycle profile and percentage of apoptotic cells, subG1 population) in propidium iodide stained cells and analysis of the mitochondrial membrane potential using JC-1 dye were performed on a FACScalibur flow cytometer (BD Biosciences, Waltham, MA, USA) as previously described [35].

### 4.12. Statistical Analysis

All quantitative data are presented as mean ± SEM. For comparisons of means, one-way ANOVA followed by Newman–Keuls test was used. Statistical computations were performed using STATISTICA 7.1 (TIBCO Software Inc., Palo Alto, CA, USA) or GraphPad Prism 6. Differences were considered statistically significant for *p* values < 0.05.

## 5. Conclusions

Our results show that sensitivity of human glioblastoma cells to synthetic cannabinoids depends primarily on the levels of cannabinoid receptor expression. The abundance of the CB2 receptors detected in tumor samples and in glioblastoma cells paves way to the use of CB2-selective, non-psychodysleptic compounds in the treatment of a large subset of gliomas. We provided a novel information about the mechanistic insights of cell death and contribution of autophagy as a cellular self-protective mechanism. We found that alterations in the *TP53* and *PTEN* genes, very frequent in gliomas, do not protect glioma cells from cannabinoid-induced cytotoxicity. The potency of synthetic cannabinoids to induce apoptosis in human glioblastoma cells despite drug resistance mechanisms caused by these genetic alterations is of great importance for the therapy of gliomas and other cancers. Combination anti-cancer therapies involving both induction or inhibition of autophagy are under investigation. In contrast to previous studies in glioma cells, our study suggests that autophagy induced by cannabinoid treatment is a protective mechanism and autophagy inhibitors may be potential agents to enhance cannabinoid action.

## Figures and Tables

**Table 1:** EC_50_s of synthetic cannabinoids estimated in glioma cell lines after 48 h treatment.

Cell Line	JWH133 (µM)	WIN55,212-2 (µM)
LN18	13.82	9.65
T98G	12.15	8.61
U87MG	18.78	9.86
U251MG	32.94	7.36
LN229	143.20	15.70

**Table 2:** Summary of glioma cell lines characteristics.

Cell Line	Source	*PTEN* Status	*TP53* Status
Primary culture			
T3	GBM (grade IV)	ND	mut
T10		ND	mut
Established cell line			
LN18	GBM-derived	wt	mut
T98G		mut	mut
U87MG	AA-derived	mut	wt
U251MG		mut	mut
LN229		wt	mut *

ND*—*not determined; *—p53 activity is partially retained.

## Data Availability

The data presented in this study are available on request from the corresponding author.

## Supplementary Materials

The following are available online at https://www.mdpi.com/2072-6694/13/3/419/s1, Figure S1: Accumulation of mutant p53 in glioma cells, Figure S2: Time-dependent effects of synthetic cannabinoids on dissipation of mitochondrial membrane potential and caspase cascade activity, Figure S3: Induction of acidic vesicular organelles (AVOs) by cannabinoids in glioma cells, Figure S4: Variability of response to the induction of autophagy in T98G, U87MG and LN229 glioma cells exposed to cannabinoids. Additional supplemental material: original scans of the immunoblots and a blank copy of informed consent.
