# Supplementary material for: Synthetic Cannabinoids Induce Autophagy and Mitochondrial Apoptotic Pathways in Human Glioblastoma Cells Independently of Deficiency in TP53 or PTEN Tumor Suppressors
Source: Cancers (Basel). 2021 Jan 22;13(3):419. doi: 10.3390/cancers13030419 (PMC7865605; doi:10.3390/cancers13030419)
Supplement: Supplementary file 1 [file cancers-13-00419-s001.zip › revised supp/Ellert_Miklaszewska et al Supplementary Information v220121 AEM final.docx]

**Supplementary Information**

Synthetic cannabinoids induce autophagy and mitochondrial apoptotic pathways in human glioblastoma cells independently of deficiency in *TP53* or *PTEN* tumor suppressors

Aleksandra Ellert-Miklaszewska, Iwona Anna Ciechomska and Bozena Kaminska


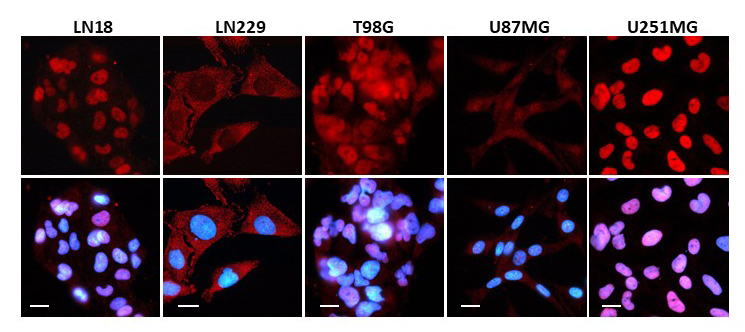


**Figure S1.** Accumulation of mutant p53 in glioma cells. Representative microphotographs of glioma cells after immunostaining for p53 (red) and counterstain for nuclei with DAPI. Note the accumulation of p53 in nuclei of LN18, T98G and U251MG glioma cells. A scale bar corresponds to 20 µm.


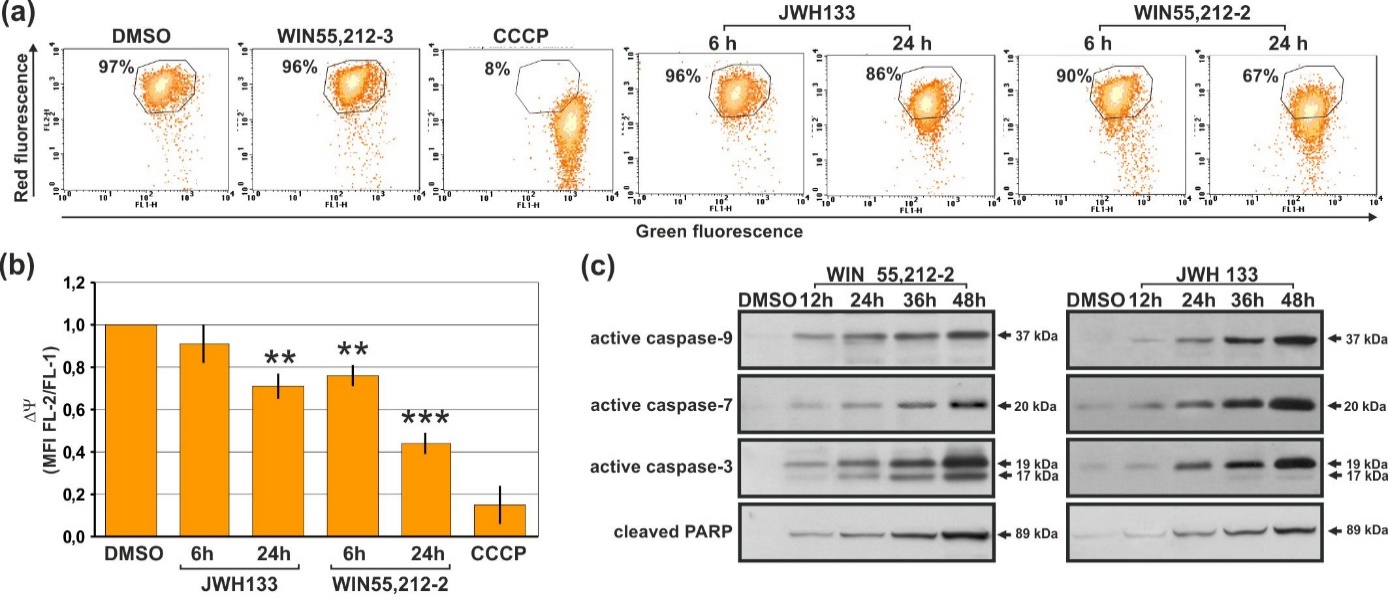


**Figure S2.** Time-dependent effects of synthetic cannabinoids on dissipation of mitochondrial membrane potential and caspase cascade activity. Flow cytometric analysis of mitochondrial transmembrane potential (*∆Ψ*) was performed in control and cannabinoid treated cells stained with the fluorescent probe JC-1. (**a**) Representative plots from FACS analysis. Percentage of cells with unchanged *∆Ψ* is shown by the gate. (**b**) A decrease of the red (FL-2 channel)/green (FL-1 channel) fluorescence intensity ratio, normalized to the values from untreated controls, corresponds to a loss of mitochondrial membrane potential. Statistical significance of changes 6h and 24 h after exposure to WIN55,212-2 or JWH133 as compared to control cells (DMSO) is indicated as follows: ** p<0.01, *** p<0.001. The inactive enantiomer WIN55,212-3 (SWIN) did not induce changes in ∆Ψ. CCCP was used as a positive control. (**c**) Synthetic cannabinoids-induced activation of caspase 9 and caspase 3, as indicated by immunodetection of cleaved proteins, was evaluated at 12–48 h post-treatment. Caspase 3 activation was also evidenced by PARP proteolysis. Equal loading of proteins was ensured by β-Actin immunodetection.


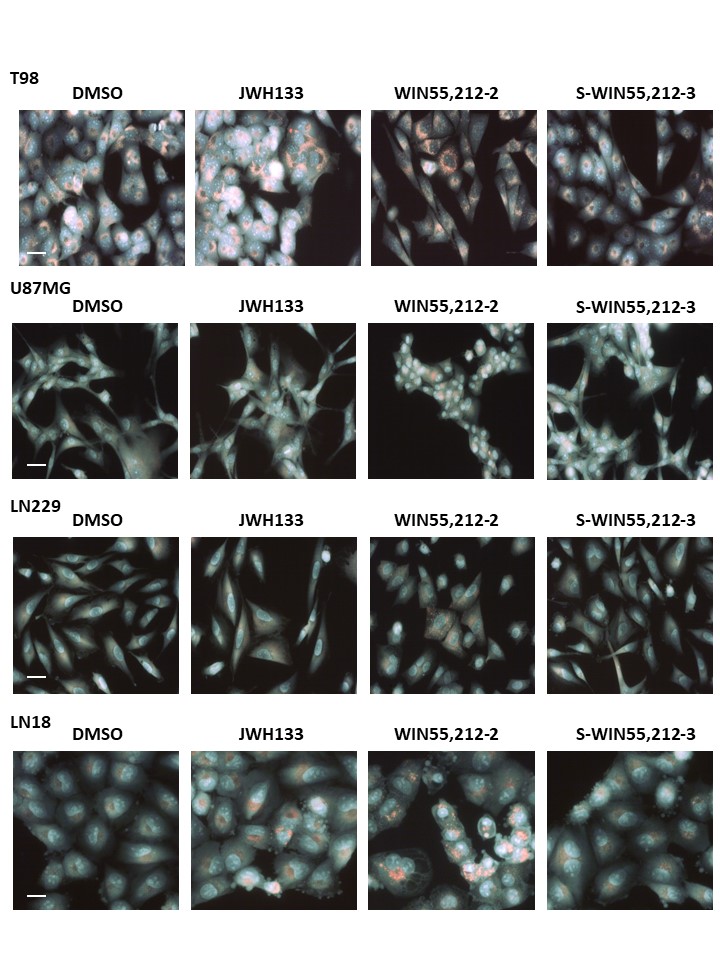


**Figure S3.** Induction of acidic vesicular organelles (AVOs) by cannabinoids in glioma cells. Glioma cells were treated with DMSO, JWH133, WIN55,212-2 or its inactive enantiomer WIN55,212-3 for 24 h and stained with acridine orange (1 µg/Ml for 15 min). Fluorescent micrographs show the formation of acidic vesicular organelles (AVOs) visible as increased red fluorescence in cannabinoid treated cells but not in control conditions. Note low response of LN229 to JWH133 and U87 to both cannabinoids. Representative results of three independent experiments are shown. A scale bar corresponds to 20 µm.


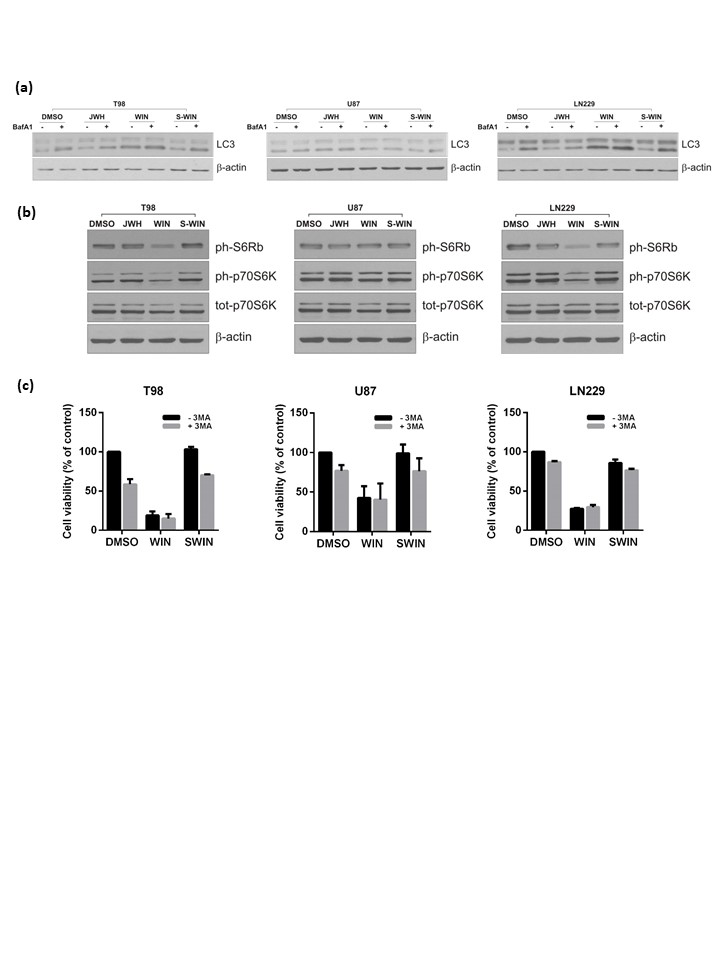


**Figure S4.** Variability of response to the induction of autophagy in T98G, U87MG and LN229 glioma cells exposed to cannabinoids. Glioma cells were treated with DMSO, JWH133, WIN55,212-2 or WIN55,212-3 (SWIN) (**a**) Representative immunoblots show LC3 levels in glioma cells 24 h after exposure to cannabinoids and after co-incubation with 10 nM bafilomycin A1 (BafA1) for the last 4 h. Similar results were obtained in two other independent experiments. (b) Representative immunoblots show the effects of cannabinoids on the phosphorylation level of mTORC1 downstream substrate: p70 S6 kinase and its target S6 ribosomal protein. Equal loading of proteins was ensured by total p70 S6 kinase and β-Actin immunodetection (**c**) Viability of glioma cells measured by MTT metabolism test after 48 h-treatment with DMSO, WIN55,212-2 or WIN55,212-3 (SWIN) and with or without addition of 3-methyladenine (3MA, 2 mM). Values were normalized to DMSO-treated cells. Results shown represent mean ± SD from three independent experiments. No significant differences in the response of cells to WIN55,212-2 were found between cells with and without 3MA co-treatment (ANOVA).
