# Supplementary figures and images for: Synthetic Cannabinoids Induce Autophagy and Mitochondrial Apoptotic Pathways in Human Glioblastoma Cells Independently of Deficiency in TP53 or PTEN Tumor Suppressors
Source: Cancers (Basel). 2021 Jan 22;13(3):419. doi: 10.3390/cancers13030419 (PMC7865605; doi:10.3390/cancers13030419)

Figure 4b

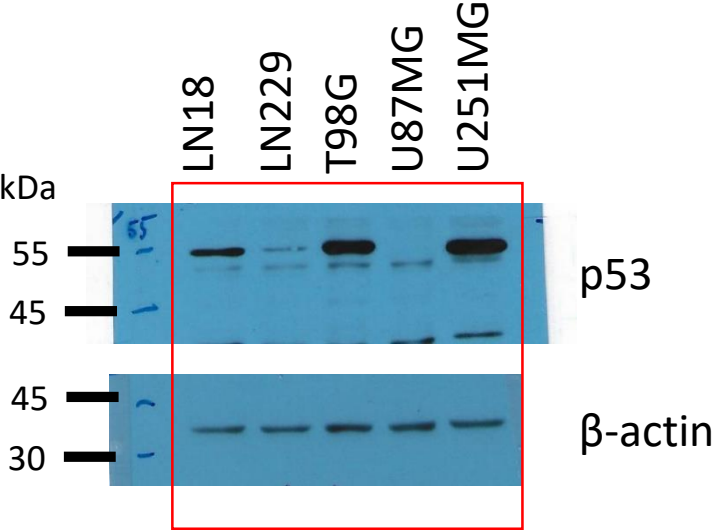

Figure 5b

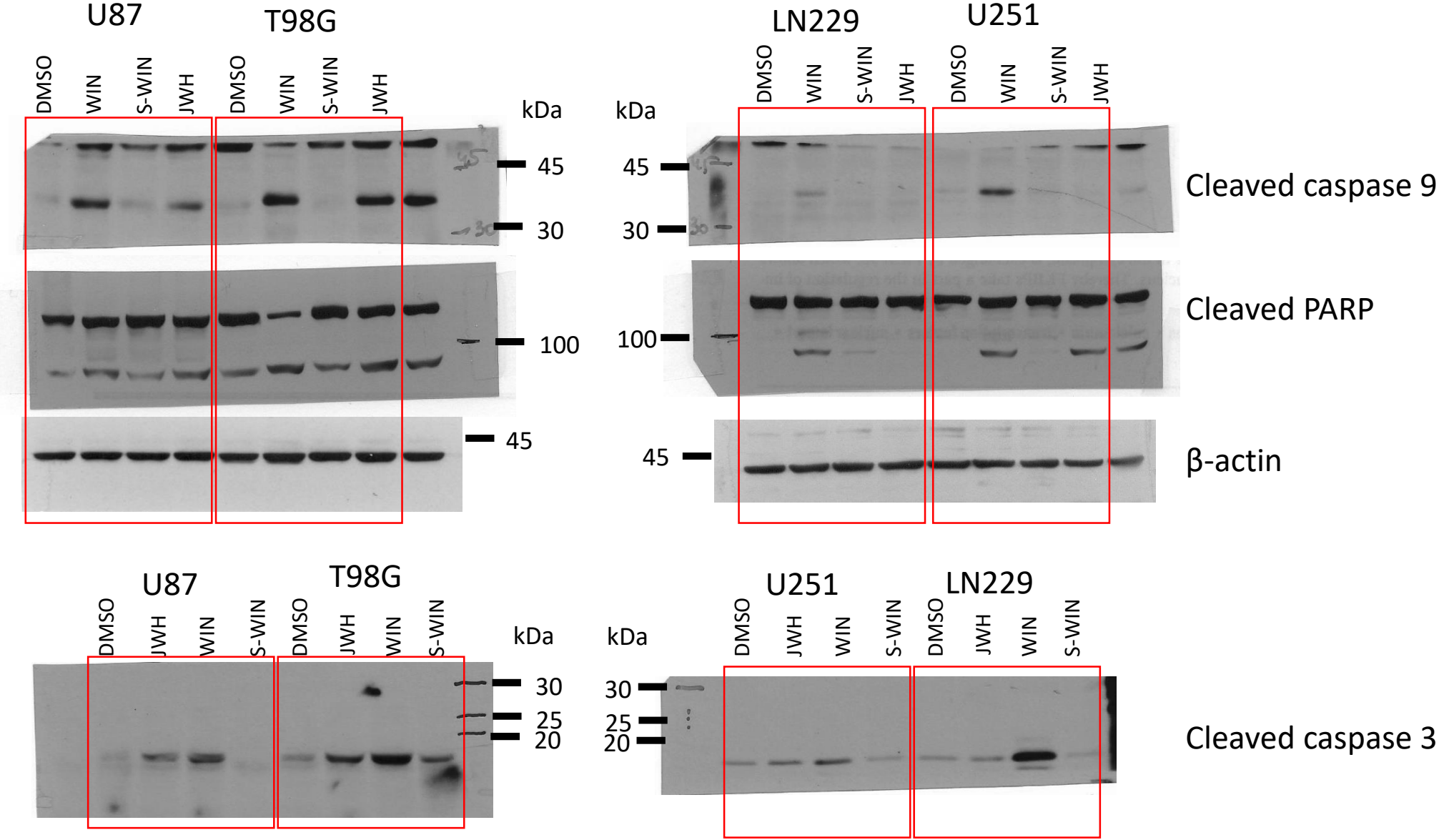

Figure 6d/e

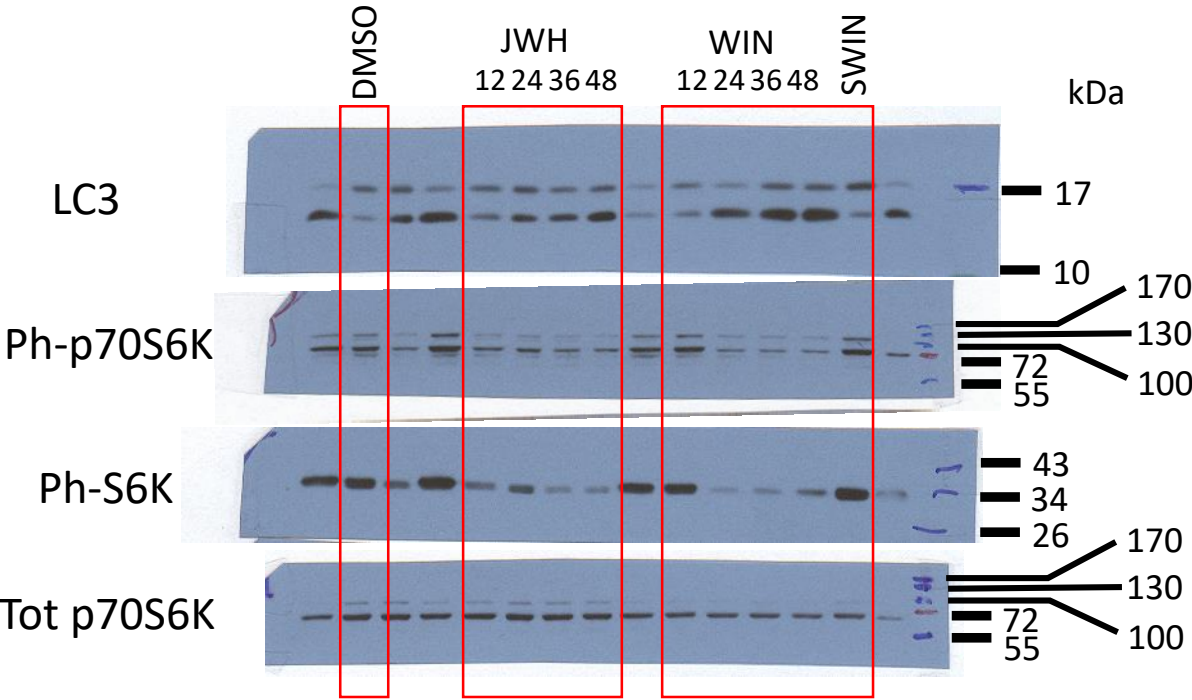

Figure 7

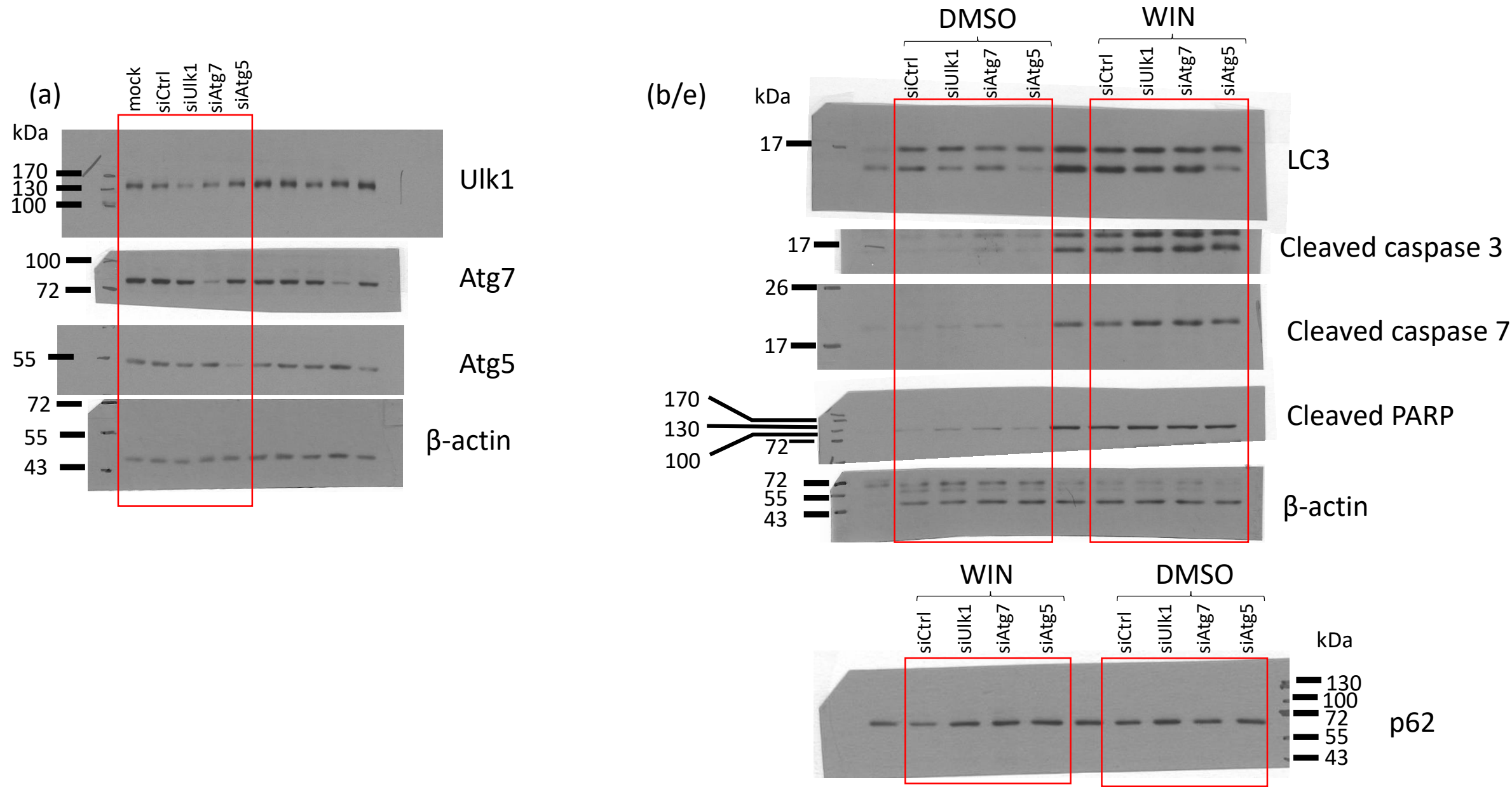

Suppl Figure 4

(a)

T98G

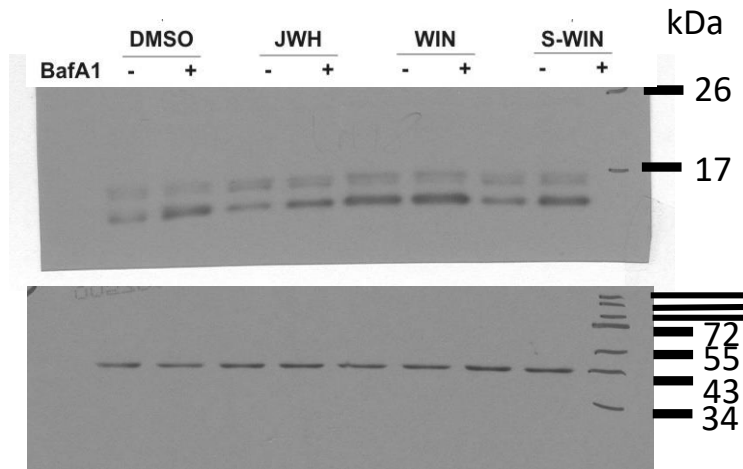

U87

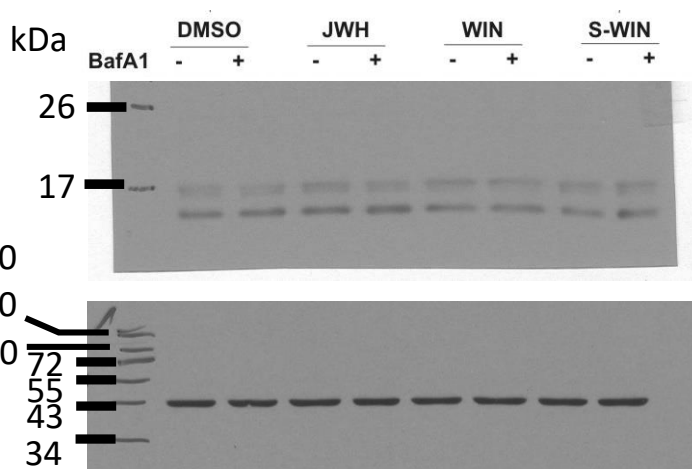

LN229

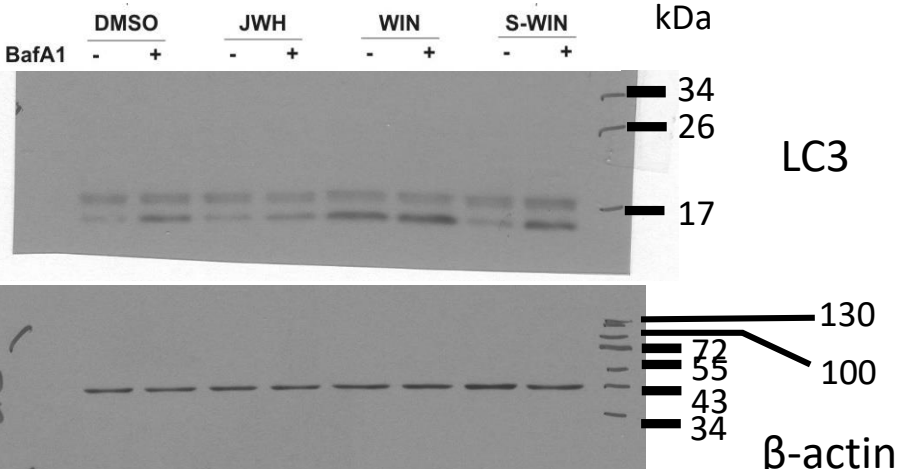

LC3

$\beta$ -actin

Suppl Figure 4 (continued)

(b)

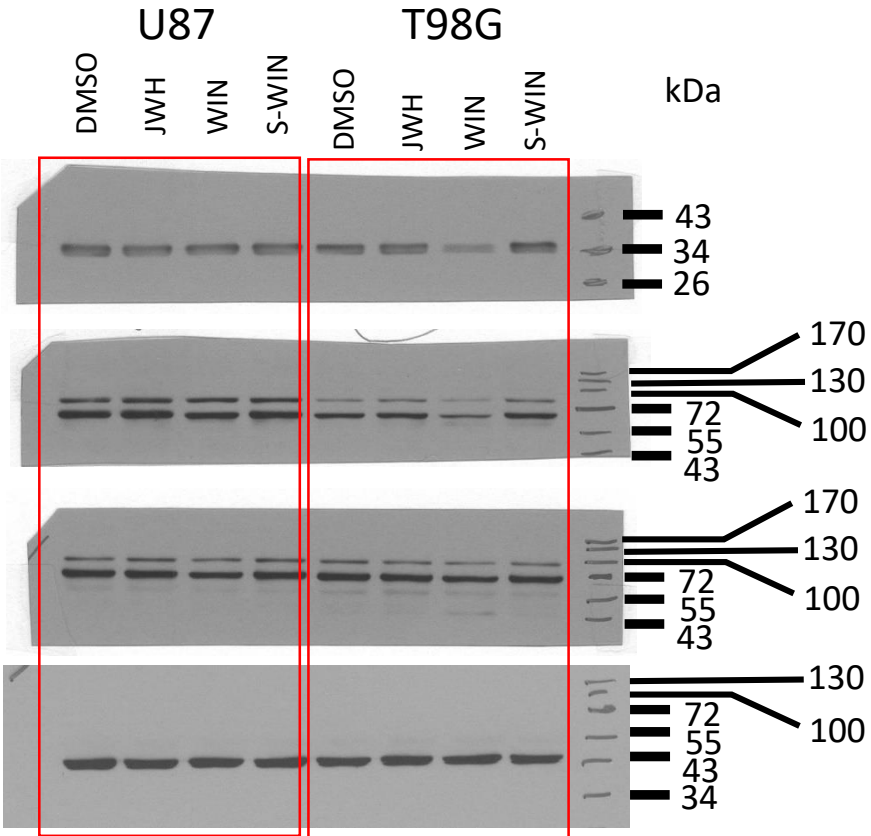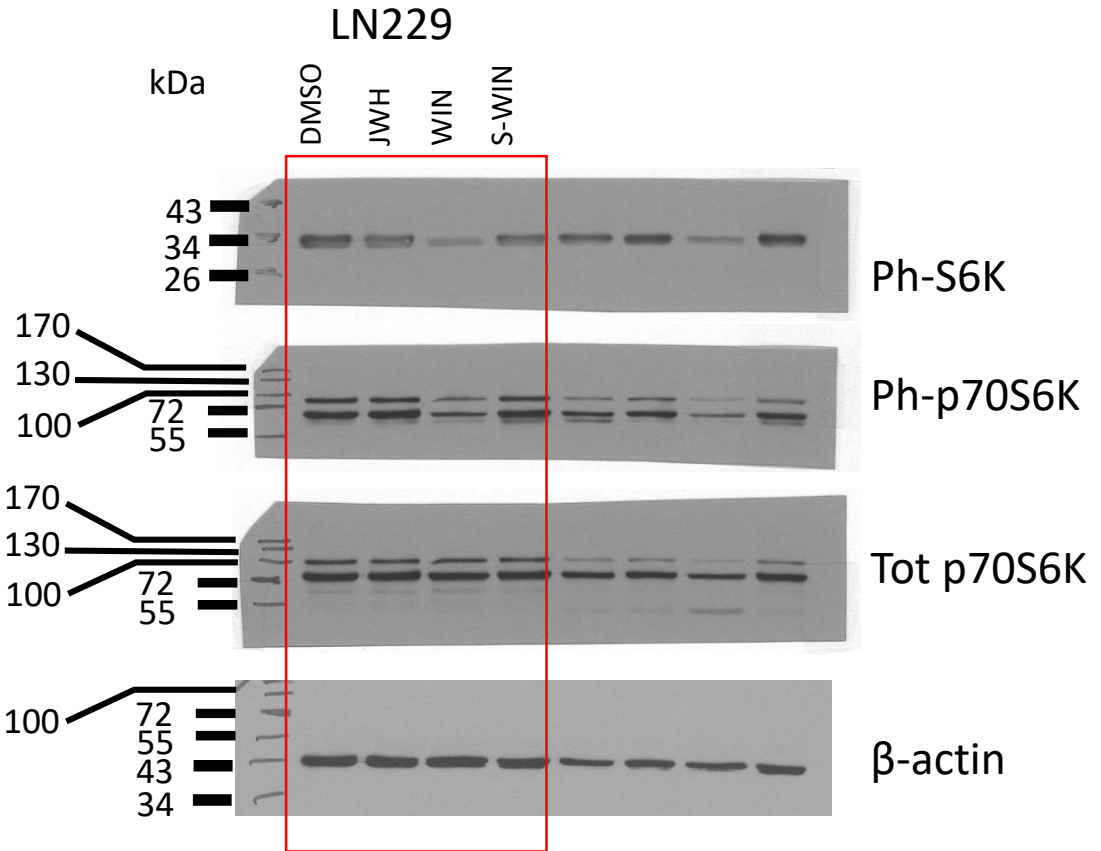

Supplement: Supplementary file 1 [file cancers-13-00419-s001.zip › revised supp/Supplemental Material original blots v14012021.pdf]
